# Diode Laser with Scaling and Root Planing for Treating Generalized Periodontitis: Case Report and Analysis of the Relevant Literature

**DOI:** 10.3390/reports7040109

**Published:** 2024-12-05

**Authors:** Teodora Tene, Anca Maria Fratila, Vasile Calin Arcas, Mihai Sava, Corina Roman-Filip

**Affiliations:** 1 Doctoral School, Faculty of Medicine, Lucian Blaga University of Sibiu, 550169 Sibiu, Romania; teodora.h.crisan@gmail.com (T.T.); calin.arcas@ulbsibiu.ro (V.C.A.); 2 Faculty of Medicine, Lucian Blaga University of Sibiu, 550169 Sibiu, Romania; mihai.sava@ulbsibiu.ro (M.S.); corina.roman@ulbsibiu.ro (C.R.-F.); 3 Military Clinical Emergency Hospital of Sibiu, 550024 Sibiu, Romania

**Keywords:** diode laser therapy, periodontal disease, scaling and root planing

## Abstract

This study evaluates the effectiveness of diode laser therapy, specifically the Biolase Epic X at 940 nm and 0.8–1 W, in conjunction with scaling and root planing (SRP) for treating generalized periodontitis. **Background and Clinical Significance**: A 32-year-old man underwent full-mouth disinfection and laser-assisted periodontal therapy, with follow-up at six months. **Case Presentation**: Significant improvements were observed, including reductions in bleeding on probing from 20% to 5%, in mean probing depth from 2.3 mm to 2.1 mm, and in clinical attachment level from −2.8 mm to −2.2 mm. Radiographic analysis showed a stabilization of bone loss and an 80% improvement in pathological sites. **Conclusions**: These findings indicate that diode laser therapy is an effective adjunct to SRP, enhancing periodontal health outcomes with minimal post-operative complications.

## 1. Introduction and Clinical Significance

Periodontal disease (PD) is triggered by factors like the buildup of plaque or tartar and the accumulation of bacteria in the periodontal pockets. These factors lead to an inflammatory response in the immune system of the host [1,2,3,4,5]. This immunological reaction results in the degradation of periodontal structures, the resorption of osseous tissue and increased tooth mobility, the recession of the gingiva, and the loss of both bone and dentition [2,3,6,7,8,9,10,11], consequently affecting oral health and general well-being [3,12,13,14].

The primary cause of the formation of periodontal pockets is the colonization of pathogenic bacteria in the subgingival biofilm [6,15,16,17], including species such as Porphyromonas gingivalis, Tannerella forsythia, Treponema denticola, and Aggregatibacter actinomycetemcomitans [13,18,19,20,21], which trigger both innate and adaptive immune responses [22,23].

Bacterial components, particularly lipopolysaccharides (LPSs), play a crucial role in initiating inflammation by activating host immune cells. This activation results in the release of proinflammatory cytokines like IL-1 and TNF-alpha, which contribute to tissue destruction and the development of periodontal pockets [24]. The inflammatory environment further exacerbates tissue damage through the activation of matrix metalloproteinases (MMPs), which degrade collagen and deepen periodontal pockets [24].

This systemic inflammatory response is supported by findings that show an increased presence of Th17 cells, particularly those expressing the IL-23 receptor, in patients with generalized chronic periodontitis, even in the absence of other systemic inflammatory diseases [21]. Moreover, periodontitis has been associated with an increased serum level of high-sensitivity C-reactive protein (hs-CRP), a marker of systemic inflammation [3,25].

In addition to bacterial factors, local irritation from dental caries and other irritative actions may contribute to PD [26]. Recent studies such as those of Chandran and Vesna [24,27] also highlight the role of viruses in PD, suggesting that viral presence in the periodontal environment may influence disease progression. This viral aspect, alongside bacterial colonization, forms a bacterial–viral model of PD, indicating that factors beyond bacteria alone are significant in its pathogenesis [27].

The host’s immune responses, both innate and adaptive, represent a critical determinant of disease progression [19,24]. The balance between bone-forming and bone-resorbing cells is influenced by immune-derived cytokines, which can either mitigate or exacerbate periodontal breakdown [6,7]. The relationship between systemic and localized inflammation, such as gingivitis and PD, and the elevation of inflammatory markers like C-reactive protein, interleukins (IL-2, IL-6), neutrophils, total white blood cells, and coagulation factors is well documented in the literature [22,23,28].

The implications of PD extend beyond esthetics, as it is associated with systemic conditions like cardiovascular disease, rheumatoid arthritis, and diabetes, as the inflammation from the periodontal pocket often maintains systemic inflammation, which is a well known part of this condition [1,3,25,28]. This underscores the importance of early diagnosis and comprehensive management strategies to mitigate its effects and improve overall health outcomes [8,9,29,30].

The effective and comprehensive management of PD necessitates a multifaceted therapeutic approach that includes both non-surgical and surgical treatments, often supplemented by adjunctive therapies [12,14,15,20,30,31,32,33,34,35,36,37,38].

Non-surgical treatment focuses on mechanical debridement through SRP to remove supra and subgingival plaque and calculus, combined with oral hygiene education to reduce periodontal inflammation [14,15,27,31,33,35,39,40]. To enhance the effectiveness of SRP, adjunctive therapies such as probiotics, paraprobiotics, and postbiotics are employed [5,35]. These agents represent innovative approaches in the management of periodontal pockets and help to modulate the immune response, reduce inflammation, and prevent bacterial colonization, thereby supporting tissue regeneration and maintaining oral health [14,35].

Additionally, ozonized materials, known for their potent antibacterial and anti-inflammatory properties, are used to irrigate periodontal pockets and promote healing [14]. Local drug delivery systems are a promising therapeutic approach in PD, offering greater efficacy and fewer adverse effects by controlling drug release; they are beneficial for diabetic patients with periodontitis as they minimize the risk of drug interactions associated with systemic administration and offer targeted therapy [41]. Immunomodulatory therapy, such as exploring the oral biofilm genome at an individual level, involves targeting the dysbiotic biofilm and modulating the host inflammatory response to reduce disease progression and tissue destruction [35,42].

Furthermore, localized antibiotic therapy can be used in association with laser therapy to target specific deep pockets, providing a more comprehensive approach to managing chronic periodontitis [13,15,37].

In cases where non-surgical methods are insufficient, particularly with significant bone loss or deep periodontal pockets, surgical interventions are necessary. These include procedures like access flaps, bone grafting, and guided tissue regeneration, aimed at reducing pocket depth and regenerating lost tissues [20,30,43].

The use of diode lasers as an adjunct to SRP has shown promise in enhancing treatment outcomes by reducing bacterial load and inflammation and promoting wound healing through photobiomodulation [20,32,36]. Lasers can effectively remove microbial species and modulate inflammatory responses, making them a valuable tool in periodontal therapy, especially for patients who are not candidates for surgical interventions [15,20,31]. Biolase Epic X is a diode laser specifically intended for soft tissue therapy that is used in a number of dental procedures, especially those intended for antimicrobial periodontal therapy [15,40]. The Biolase Epic X diode laser, primarily known for its application in dental bleaching [44,45], has potential implications for PD management. While the primary focus of the Biolase Epic X is cosmetic, its underlying technology and the broader context of PD treatment suggest [4,13,15,16,20,31,32,46,47,48] possible therapeutic benefits.

Continuous maintenance care and monitoring of the oral microbiota are crucial in sustaining the benefits of initial treatments and preventing disease recurrence [13,14].

This comprehensive approach underscores the importance of personalized treatment plans that consider the unique microbial profiles and clinical needs of each patient, paving the way for more effective and sustainable PD treatment [14,15].

The aim of the present case report is to demonstrate the efficacy of diode laser therapy, in conjunction with SRP, in the treatment of generalized periodontitis. This case is significant as it highlights the successful outcomes achieved, including significant reductions in probing depth, bleeding on probing, and clinical attachment level, showcasing the potential of this therapy to enhance periodontal treatment results and improve patient outcomes.

## 2. Case Presentation

### 2.1. Patient Presentation

**Figure 1:**
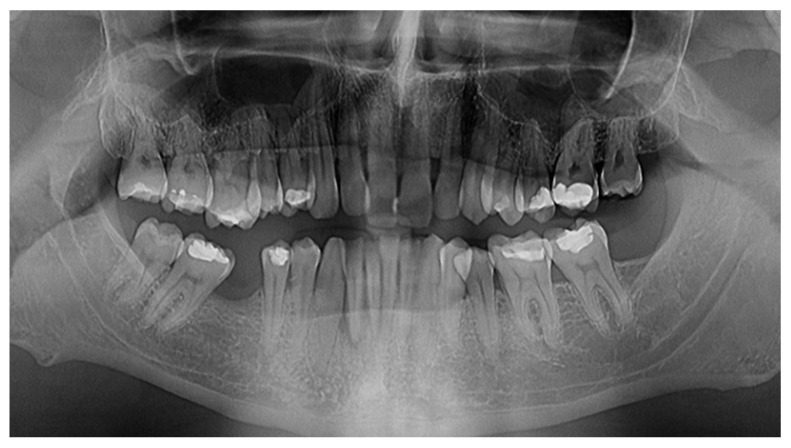
Initial dento-periodontal status—radiological view.

**Figure 2:**
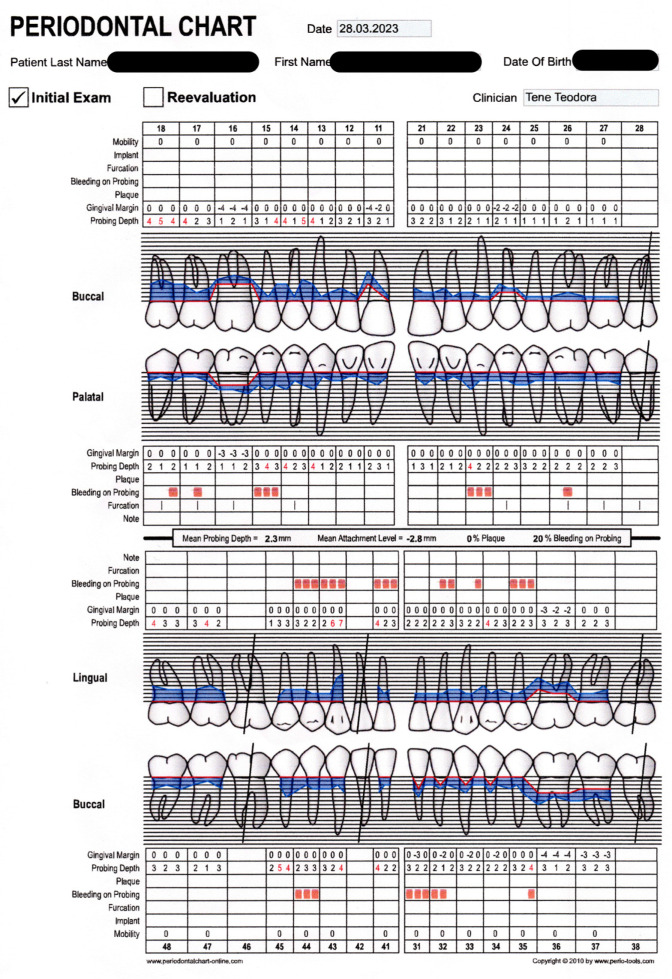
Periodontal chart—initial clinical examination. Blue outline: probing depth; red outline: gingival margin; red square: bleeding on probing; red texts: probing depths deeper than 4 mm.

Patient P.A., a 32-year-old man, presented to the Dental Ambulatory Compartment of the Military Hospital of Sibiu, with complaints of general dental sensitivity, gingival recession, and spontaneous gingival bleeding. The patient was clinically healthy with no significant medical history, was a non-smoker, and reported no use of alcohol or psychoactive substances. Upon initial consultation, a comprehensive dental evaluation was conducted, which included both radiological imaging (Figure 1) and a specific periodontal examination (Figure 2).

Figure 2 provides a lot of information about the periodontal status of the patient, in standard color code. Blue outline around the tooth highlights the probing depth, while the red one refers to gingival retraction. Probing depths deeper than 4mm are coloured in red, which means it has a pathological value.

The periodontal chart evaluates the mobility of the teeth, furcation, presence/absence of implants/dental plaque, and bleeding on probing. The patient presented does not have implants, dental plaque, teeth mobility or furcation damage. Bleeding on probing was checked in same 6 points as the probing depth and recorded where found.

### 2.2. Initial Consultation

The periodontal assessment involved measuring the probing pocket depth (PPD). The periodontal probe was used by gently inserting it into the gingival sulcus at six specific points on each tooth: the disto-vestibular, centro-vestibular, mesio-vestibular, disto-oral, centro-oral, and mesio-oral points. The clinician carefully inserted the probe until resistance was felt at the base of the pocket, which corresponded to the periodontal attachment. The depth of the pocket was measured by reading the millimeter markings on the probe where the gingival margin met the probe. This process was repeated for each tooth, and the measurements were recorded in the periodontal chart. Care was taken to apply gentle pressure to avoid causing discomfort or trauma to the tissues while obtaining accurate readings. These measurements were recorded on the periodontal chart evaluation sheet [49]. All the measurements were performed by the same clinician.

Radiological examination (Figure 1) was performed prior to treatment using digital imaging, obtaining a panoramic image, 0.025 mSv, which provided a clear assessment of the extent of bone resorption and the depth of periodontal pockets and the shape and height of the alveolar crest and furcation defects. The type of bone loss (horizontal or vertical) is a very important aspect that might indicate the appropriate treatment approach [11]. These images served as both a diagnostic tool and a baseline for comparison during follow-up evaluations (Figure 2).

Inflammation was assessed using the bleeding on probing (BOP) index and the presence of plaque during the initial periodontal evaluation. The bleeding on probing (BOP) index was determined by gently probing the periodontal pockets using a periodontal probe at six points around each tooth, as described earlier. After the probing, the presence or absence of bleeding was noted. Bleeding is an indicator of gingival inflammation, as healthy gums typically do not bleed when probed. The percentage of sites that bled upon probing was calculated, with higher percentages indicating greater levels of inflammation.

Plaque was also assessed during the periodontal exam, as it can indicate a possible cause of the periodontal tissue inflammation.

Radiographic (Figure 1) evaluation revealed both horizontal and localized vertical bone resorption, particularly around tooth 4.2, which demonstrated grade-three mobility, indicating the need for extraction. Figure 1 shows the initial dento-periodontal status: it presents the mean probing depth, which is 2.3 mm, the mean attachment level of 2.8 mm, and 20% bleeding at probing sites, which suggests periodontal tissue inflammation. All these issues can be corrected with proper therapy (SRP and laser treatment) and good oral hygiene.

Clinical examination (Figure 2) led to the following findings: the dental site with biggest attachment loss had a loss of 3–4 mm, bone loss was extended in the coronal area, one tooth was lost due to PD, there was mostly horizontal bone loss, and the patient’s history of bone loss and his age were favorable; he was clinically healthy, was a non-smoker, and did not have any collateral known illnesses.

Based on the clinical and radiological findings, the patient was diagnosed with generalized periodontitis, stage II, grade A, according to the 2018 classification of Carranza’s Clinical Periodontology [50].

### 2.3. Treatment Plan and Rationale for Laser Therapy

After establishing the diagnosis, a treatment plan focusing on non-surgical periodontal therapy was recommended. The full-mouth disinfection (FMD) protocol was selected, which involved SRP in a closed field [50]. The decision to use laser therapy, rather than adjunctive therapies such as systemic antibiotics or local drug delivery systems, was based on the laser’s ability to precisely target and reduce periodontal pathogens, promote faster tissue healing and avoiding the risk of antibiotic resistance. Additionally, laser therapy offers a non-invasive, painless alternative that accelerates recovery and reduces patient discomfort. The Biolase Epic X diode laser was chosen for its established efficacy in periodontal pocket sterilization, operating at a wavelength of 940 nm, which is optimal for bacterial reduction and tissue healing.

### 2.4. Procedure Protocol

Before beginning the procedure, the patient was prepared with local anesthesia, which was necessary to ensure comfort during both the scaling and the laser treatment; a 1.8 mL ampoule of Articaine 3% 1:200,000 was used. The patient was also given oral hygiene instructions to reinforce the importance of maintaining daily plaque control in managing PD.

The SRP procedure was conducted in a closed field, using a combination of ultrasonic scalers and hand instruments, specifically, Hu-Friedy Gracey curettes, in order to thoroughly debride the periodontal pockets. Curettes no. 1/2 and no. 5/6 were used for the frontal teeth (upper and lower incisors and canines—vestibular, distal, mesial, and oral surfaces), curettes no. 7/8 were used for all lateral teeth (from premolar to molar and also vestibular and oral surfaces, both maxillary and mandibular), and no. 11/12 and 15/16 instruments were used for all mesial surfaces from the first premolar to the last molar, both maxillary and mandibular. Curettes no. 13/14 and 17/18 were used for the distal surfaces on the lateral teeth (from the first premolar to the last molar in the upper and lower arch). All supra- and subgingival plaque and calculus were removed to reduce the bacterial load in each pocket. Once the mechanical debridement was complete, the root surfaces of the affected teeth were carefully smoothed to prevent further bacterial accumulation and promote the reattachment of the gum tissue using an erythritol powder profijet. During the SRP procedure, special attention was paid to the deep periodontal pockets, particularly around tooth 4.2, which showed localized vertical bone resorption and grade-three mobility. The decontamination process was supplemented with the irrigation of periodontal pockets using a 2% Chlorhexidine solution, further reducing bacterial load and promoting an optimal environment for healing.

Following SRP, the Biolase Epic X diode laser was applied for periodontal pocket sterilization. The laser was set to a power of 0.8–1 W in continuous wave mode. Each periodontal pocket, regardless of depth, was treated with the laser to eliminate residual bacteria and promote tissue healing. The laser treatment was completed in a single session. The choice of the 940 nm wavelength was based on its effectiveness in targeting periodontal pathogens while promoting faster tissue recovery and minimizing damage to surrounding healthy tissues.

Post-treatment care instructions were provided to the patient, including the use of a chlorhexidine mouth rinse twice daily for one week, along with continued strict oral hygiene practices to maintain the results of the treatment.

### 2.5. Six-Month Follow-Up and Evaluations

**Figure 3:**
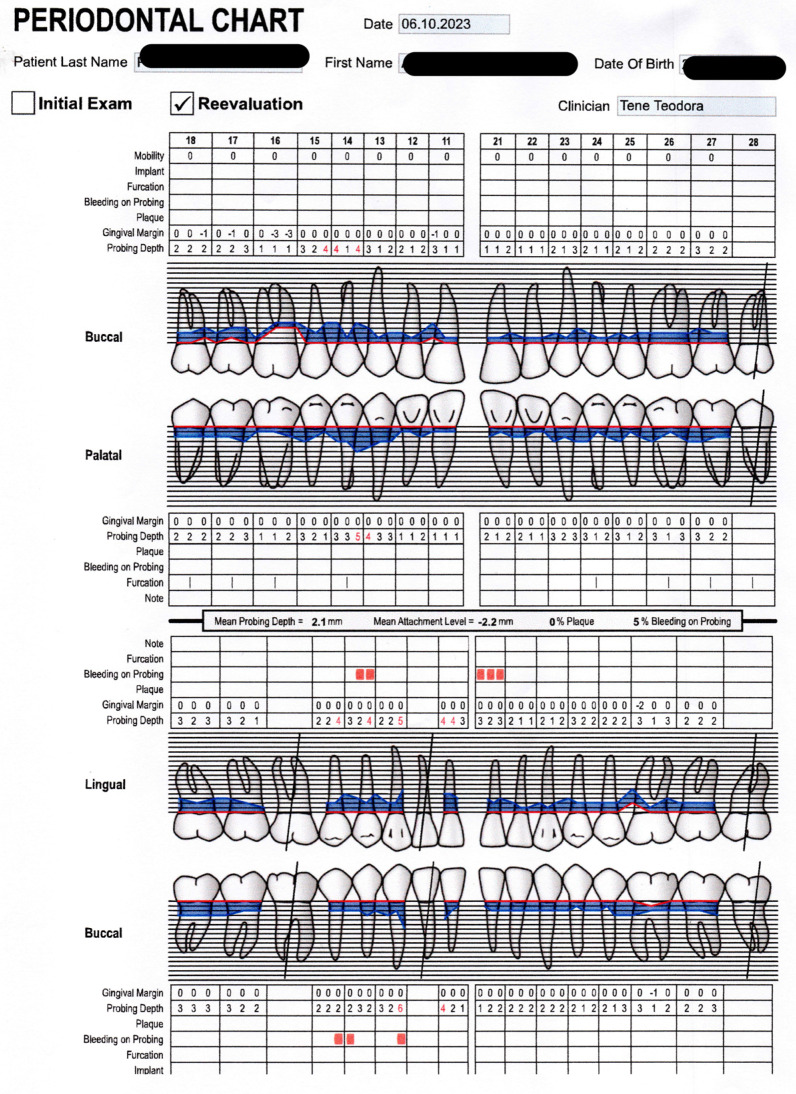
Periodontal reevaluation. Blue outline: probing depth; red outline: gingival margin; red square: bleeding on probing; red texts: probing depths deeper than 4 mm.

**Figure 4:**
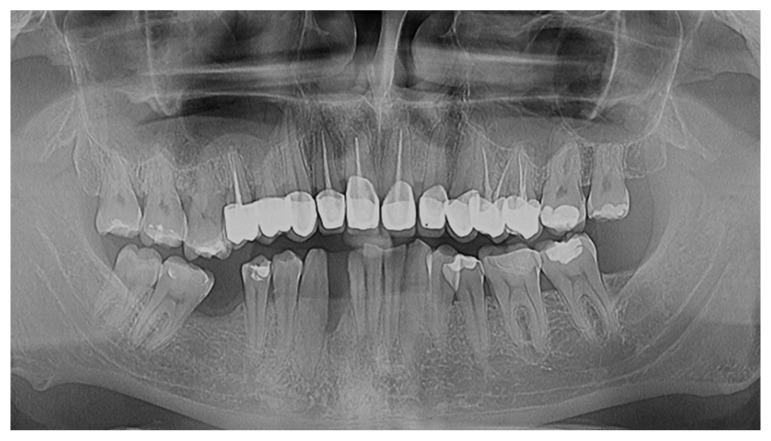
The dento-periodontal status—radiological view—follow-up—one year.

At the six-month follow-up, the patient underwent a comprehensive clinical and radiological re-evaluation. Clinically, significant improvements were noted. Bleeding on probing had decreased, as well as the main probing depth, which was reduced from 2.3 mm to 2.1 mm; the main clinical attachment level had also improved, as can be seen from the second periodontal chart (Figure 3) and the follow-up radiological image (Figure 4). Radiological analysis revealed the stabilization of the previously observed bone resorption, with no further progression of PD.

The periodontal chart showed an improvement in the periodontal status of the patient: the main probing depth was 2.1 mm, bleeding on probing was reduced, as well, and the clinical attachment loss had decreased, which is the best sign of healthier periodontal tissue. The therapy showed its benefits both in the PD markers and in reducing tooth sensitivity.

As we can see in Figure 3, on the graphics, blue outline (probing depth), red outline (gingival margin) are visibly reduced. Also, bleeding on probing (red square next to the point where found) had decreased. 

The combined use of laser therapy and SRP resulted in notable clinical and radiological improvements, underscoring the effectiveness of this treatment approach for moderate PD.

Following the completion of the SRP procedure combined with laser-assisted periodontal therapy, significant improvements were observed in the patient’s periodontal health over the course of six months. Clinically, bleeding on probing (BOP) decreased from an initial 20% to 5%, indicating a marked reduction in inflammation. Additionally, the mean probing depth across all periodontal pockets reduced from 2.3 mm to 2.1 mm, while the mean clinical attachment level improved from −2.8 mm to −2.2 mm. These improvements in clinical parameters were significant, reflecting the success of the combined treatment.

Radiological evaluation also confirmed the positive clinical outcomes. Initial radiographs had revealed both horizontal and localized vertical bone resorption, particularly affecting tooth 4.2, which exhibited grade-three mobility. At the six-month follow-up, the radiographic analysis showed the stabilization of bone loss, with no further progression in resorption. This radiological stability, combined with the clinical improvements, underscores the effectiveness of SRP and laser therapy in controlling the progression of periodontitis and maintaining periodontal health.

**Figure 5:**
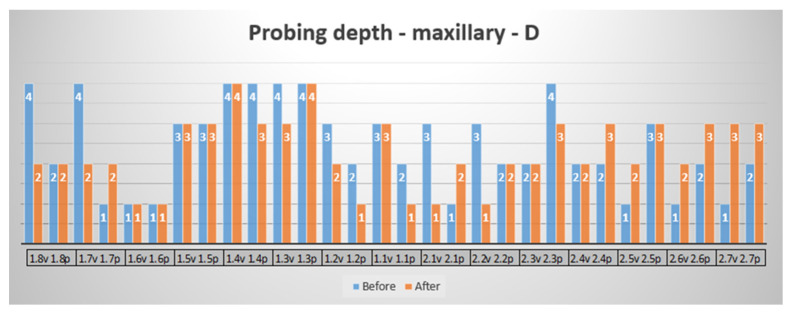
Probing depth—maxillary—vestibular surface, distal probing point.

Furthermore, more than 80% of the periodontal pockets that initially exhibited pathological depths showed a significant improvement or remained stable. Only one site presented a minor increase in pocket depth, from 3 mm to 5 mm, which remained under clinical control. The treatment resulted in better attachment levels and healthier gingival tissue overall (a healthy color and texture), with no signs of adverse effects such as excessive sensitivity or discomfort post-treatment. Figure 5 shows a decrease of pocket depths, or remained unchanged, but very important no increasing of the pathological values. Few increasing values (from 1 to 2 mm at teeth 2.5, 2.6, 2.7 and from 2 to 3 mm at Tooth 2.7 palatal) are not pathological. These findings demonstrate the success of the combined SRP and laser therapy approach in managing moderate PD and improving the patient’s overall periodontal condition.

In the SRP and laser periodontal evaluations, a certain gingival site with significant improvement was observed, as well as sites where the status remained the same and four sites where the probing depth was 1 mm greater than it initially was. None of the sites with an unfavorable initial status (greater than 4 mm) presented a negative change; the probing depth was reduced (healthy gingival tissue) or remained the same (the disease did not progress).

**Figure 6:**
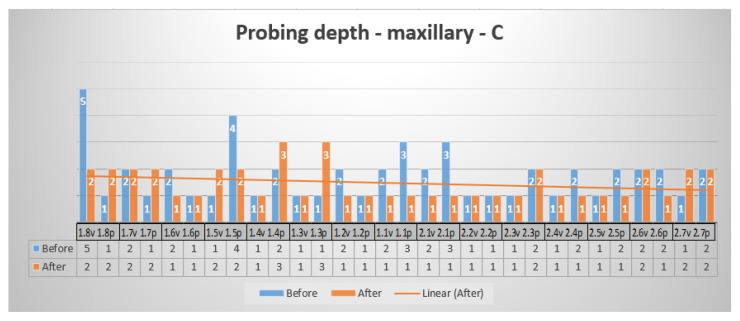
Probing depth—maxillary—vestibular surface, central probing point.

Figure 6 shows a comparison, before and after periodontal treatment, of the vestibular surface of maxillary teeth with the central probing point. Two of the probing values were pathological (5 and 4), both placed in the lateral area. After surgical SRP treatment and laser, the probing values became normal, with healthy tissue.

**Figure 7:**
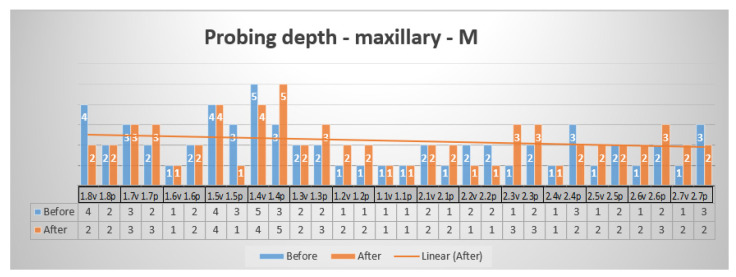
Probing depth—maxillary—vestibular surface, mesial probing point.

The three values greater than the physiological ones in the initial exam—the vestibular surface and the mesial surfaces of the superior teeth presented in Figure 7—were reduced after periodontal treatment. The physiological values (1–3 mm probing depth) did not present significant change. One dental site presented a slight increase (from 3 mm to 5 mm).

**Figure 8:**
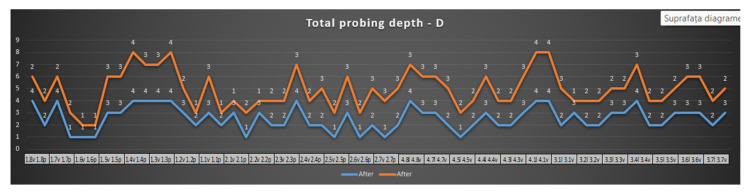
Probing depth—maxillary and mandibular—distal surface.

In Figure 8, a tendency of stabilization of periodontal pockets can be observed. The number of periodontal pockets with pathological values was significantly reduced after the SRP and laser treatment. Out of the 11 sites with pathological values, 7 improved after treatment (63% of the pathological sites presented physiological values), which means more than half of them presented improvements.

**Figure 9:**
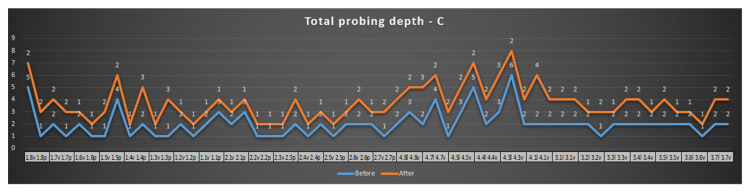
Probing depth—maxillary and mandibular—central surface.

Figure 9 highlights the effects of the surgical SRP treatment and laser. From the pathological values initially evaluated, there was an improvement in 80% of the periodontal pockets.

**Figure 10:**
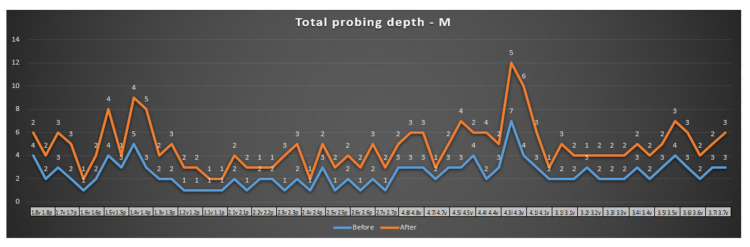
Probing depth—maxillary and mandibular—mesial surface.

In Figure 10, an improvement in the periodontal status on the mesial surface can be observed. This improvement is not as obvious as it is on the other surfaces examined. The depth of the periodontal pockets is reduced, but it is also observed that a number of them continue to be within the pathological value range (over 4 mm).

## 3. Discussion

The treatment of PD should have, as a main aim, the elimination of the bacterial load. Secondary aims should include the stimulation of gingival tissue healing, the improvement of oral hygiene, a reduction in healing time, and improvements to quality of life [13]. The use of laser therapy in PD has multiple benefits, such as accelerating the healing process, allowing painless treatment, reducing or even eliminating post-operative complications, improving quality of life, and reducing the duration of treatment [47].

A large population is currently affected by PD, and at a younger age than before. Studies show that 47% of adults aged over 30 suffer from PD. Because of this, the importance of efficient, fast, and painless treatment that stops the progression of the disease is obvious. In addition to the classic treatment (SRP), improvements to oral hygiene, and antibiotherapy, this innovative irradiation treatment with a diode laser is being used more often. In the treatment of medium or severe PD, the combined treatment of SRP + diode laser significantly improves therapy results [33]. While some authors [33] underline the importance of antibiotherapy in PD treatment, others [37] are searching for auxiliary methods, the avoidance of pharmaceutical treatment, and the use of laser therapy.

PD management requires a multi-faceted approach, and diode laser therapy as an adjunct to SRP has emerged as a promising solution [38]. In this case report, the use of diode laser therapy showed significant improvement in the patient’s periodontal health, with reduced bleeding on probing (BOP) and improved the probing depth. Similar results have been observed in other studies, where diode lasers, specifically in the 940 nm range, have been shown to enhance the clinical outcomes of non-surgical periodontal treatments by significantly reducing microbial counts and improving clinical parameters [51].

Shuchen Yu, Xiaodan Zhao et al. in their study “Clinical effectiveness of adjunctive diode laser on SRP in the treatment of periodontitis: is there an optimal combination of usage mode and application regimen? A systematic review and meta-analysis” from 2022 showed the importance of the use of the diode laser in periodontal pockets over multiple visits for obtaining the best results possible [32].

Several clinical trials have demonstrated the efficacy of diode lasers in periodontal therapy. For instance, a study by Schwarz et al. showed that diode lasers significantly reduced probing depths and bleeding in patients with chronic periodontitis compared to SRP alone [52]. Similarly, research by Giannelli et al. highlighted the enhanced healing effects of diode lasers due to their ability to ablate inflamed tissue and promote regeneration [53]. These studies align with the results observed in this case report, further confirming the benefits of using diode lasers as an adjunct to SRP.

After laser treatment, no adverse effects such as discomfort, pain, hypersensitivity, or a burning sensation have been observed, irrespective of the age, gender, or associated pathologies of the patient [4]. The literature has shown the ability of the laser wavelength to modulate the inflammatory response of the patient, significantly reducing it in a short period of time, with no pain [54].

The disinfection of periodontal pockets can be carried out efficiently, painlessly, and with no complications with laser therapy. Studies show that microbial species in the oral cavity can be eliminated through this therapy. Laser effectiveness has been proven both as a prevention method and a treatment (reducing the periodontal pockets) [48].

In contrast, other clinical trials, such as those conducted by Dengizek Eltas et al., used different laser protocols and wavelengths, such as low-level laser therapy (LLLT) and CO_2_ lasers [34,55]. Although these studies also demonstrated improvements in periodontal outcomes, the protocols and results varied slightly. The CO_2_ laser, for example, showed superior results in reducing bacterial loads but required multiple sessions for comparable results [34]. The variation in outcomes may be attributed to differences in laser wavelengths and treatment protocols, highlighting the importance of selecting the appropriate laser technology for individual cases [36].

A comparison of various laser-assisted periodontal therapies revealed that while diode lasers provide significant clinical benefits, the results are influenced by the protocol and laser settings used [56]. Studies using different wavelengths, such as the 810 nm diode laser, have shown similar reductions in probing depth but require a longer treatment course [57].

Snehal Dalvi, Namrata Khetal et al. in their study called “Utilization of 810 nm Diode Laser Treatment in Periodontitis as an Alternative to Surgical Debridement Approach” [20] showed the efficiency of the diode laser of 810 nm in chronic PD with pockets of 6–8 mm. This treatment can be used as an alternative to surgical treatment, significantly improving periodontal health. No after-treatment complications such as edema, pain, or profuse bleeding were observed. Epic X Laser is a diode laser using a 940 nm wavelength that has demonstrated benefits in PD treatment and is used in our case report. So, both wavelengths, 810 nm and 940 nm, are effective in the treatment of periodontal pockets.

In this case report, the use of a single-visit diode laser treatment with SRP proved highly effective, suggesting that the 940 nm wavelength may offer a balance between treatment efficiency and patient comfort.

The use of the current technique, as with the use of the diode laser, for treating chronic PD leads to a decrease in discomfort for the patient, a decrease in pain during the treatment, and a decrease in dental sensitivity, so it increases quality of life. Studies also show a decrease in the healing time of the tissues [47].

Despite the positive outcomes in this case, this study has limitations. One of the key limitations is that it focuses on a single patient, which limits the generalizability of the findings. Additionally, the complexity of the case was moderate, and the results may vary in patients with more severe PD. Future studies involving larger patient cohorts and varying degrees of PD severity are necessary to validate these findings.

The clinical implications of this study are notable, as the protocol used can be readily implemented in general dental practice. Diode laser therapy is a relatively simple, non-invasive adjunct to SRP that enhances the effectiveness of periodontal treatment while minimizing post-operative discomfort. Furthermore, the short treatment time and single-visit protocol make it an attractive option for both clinicians and patients.

One limitation in replicating this study is the cost associated with the diode laser used for the periodontal treatment. High-quality lasers, such as the Biolase Epic X used in this study, can be quite expensive, making it challenging for smaller or less-equipped dental practices to implement this protocol. This financial barrier may limit the accessibility of laser-assisted periodontal therapy, especially in settings where resources are more constrained, potentially reducing the feasibility of this treatment option for broader clinical use.

In terms of future research, this study provides a foundation for more detailed investigations into the use of diode lasers in periodontal therapy. As this case is part of a broader doctoral research project, future studies will explore the long-term effects of diode laser therapy, focusing on optimizing the protocol and assessing its effectiveness in more complex periodontal cases. Clinical trials involving multiple patients and varying protocols will be necessary to refine the treatment and improve outcomes further. Additionally, exploring the potential for combining diode lasers with other adjunctive therapies, such as photodynamic therapy or local drug delivery, may offer even greater clinical benefits.

## 4. Conclusions

This case report demonstrates that the integration of diode laser therapy into SRP significantly improved periodontal health in a patient diagnosed with generalized periodontitis.

Periodontal pockets deeper than 4 mm decreased in depth, with only one site showing an increase from 3 mm to 5 mm. The majority of pathological sites either improved or remained stable, confirming the effectiveness of this treatment.

Additionally, the health of the gingival tissue improved significantly, as shown by the reduction in bleeding on probing from 20% at the initial evaluation to just 5% at the recall visit. This improvement reflects the successful management of inflammation and bacterial load. The reduction in probing depth from a mean of 2.3 mm at baseline to 2.1 mm at the follow-up assessment, combined with an improvement in average attachment levels from −2.8 mm to −2.2 mm, illustrates the recovery of the periodontal tissues.

However, one limitation of this study is the high cost associated with diode laser therapy, which may limit its accessibility in routine clinical practice. Further investigation is advised to evaluate the prolonged consequences and economic efficiency of this protocol, particularly across a more extensive patient demographic and more severe periodontal instances.

## Data Availability

The data are not publicly available due to privacy concerns. The raw data supporting the conclusions of this article will be made available by the authors on request.
